# Role of the Membrane Transport Mechanism in Electrochemical Nitrogen Reduction Experiments

**DOI:** 10.3390/membranes12100969

**Published:** 2022-10-02

**Authors:** Marco Leonardi, Giuseppe Tranchida, Roberto Corso, Rachela G. Milazzo, Salvatore A. Lombardo, Stefania M. S. Privitera

**Affiliations:** 1 National Research Council Institute for Microelectronics and Microsystems (CNR-IMM), Strada VIII, 5, 95121 Catania, Italy; 2 Department of Physics and Astronomy, University of Catania, Via Santa Sofia, 64, 95123 Catania, Italy; 3 Department of Chemical Sciences, University of Catania, Viale Andrea Doria, 6, 95125 Catania, Italy

**Keywords:** Nafion, Zirfon, ammonia contamination, nitrogen reduction reaction, membrane separator

## Abstract

The electrochemical synthesis of ammonia through the nitrogen reduction reaction (NRR) is receiving much attention, since it is considered a promising alternative to the Haber–Bosch process. In NRR experiments, a Nafion membrane is generally adopted as a separator. However, its use is controversial since ammonia can be trapped in the membrane, to some extent, or even pass through it. We systematically investigate the interaction of a Nafion membrane with ammonia and with an electrolyte and compare it with Zirfon as a possible alternative separator. We show that Nafion containing ammonia can easily release it when immersed in a 0.1 M Na_2_SO_4_ ammonia-free electrolyte, due to the cation exchange mechanism (Na^+^-NH_4_^+^). Since Na_2_SO_4_ is a commonly adopted electrolyte for NRR experiments, this may cause serious measurement errors and non-reproducible results. The same experiments performed using the polysulfone Zirfon separator clearly show that it is immune to interactions with ammonia, because of its different ion conduction mechanism. The findings provide a deeper understanding of the choice of membrane and electrolyte to be adopted for NRR tests, and may allow one to obtain more accurate and reliable results.

## 1. Introduction

Ammonia is largely employed in the agriculture, chemical, pharmaceutical, food and beverage industries, and it also has great potential for hydrogen storage as an energy vector to mitigate intrinsic fluctuations of renewable energy sources. The industrial production of ammonia is carried out by the Haber–Bosch process by reacting H_2_ and N_2_ gases at high temperatures (350–550 °C) and pressures (150–350 atm). Since hydrogen is mainly obtained by steam reforming, ammonia production results in a highly polluting process that generates roughly 1–2% of total CO_2_ annual emissions [1].

Electrochemical ammonia synthesis under mild conditions and based on the nitrogen reduction reaction (NRR) [2,3] is considered an interesting strategy to store renewable energy and address the climate change emergency. Despite the great interest and the efforts that have been made to improve the technology required for the electrochemical synthesis of ammonia, this approach still faces many problems. One major issue is the lack of an efficient catalyst capable of dissociating the N_2_ triple bond and competitively producing ammonia if compared to the Haber–Bosch process. Another issue is related to the high ammonia solubility in water, in the form of NH_3_ or NH_4_^+^, and to the high interaction of ammonia with many surfaces, because of its electric dipole, leading to the necessity of adopting a rigorous protocol to evaluate the activity of the catalysts through a reliable determination of the produced ammonia. A rigorous protocol has recently been proposed to minimize the effects of various sources of environmental contamination [4,5]. The core of many electrochemical devices, such as fuel cells and electrolyzers, is the membrane electrode assembly (MEA), in which a proton exchange membrane (PEM), or an alkali anion exchange membrane (AAEM), the catalyst, and the field plate electrodes are assembled in a stack. The effect and the possible contamination of all these parts have to be taken into account. On the other hand, most research laboratories employ H-type cells to test the NRR activity of catalysts. In this cell, the counter and the working electrodes are positioned in two distinct compartments and separated by a membrane to avoid O_2_ diffusion to the cathode and NH_4_^+^ decomposition on the counter side. Nafion membranes are typically adopted [6,7,8]. However, it has been recently shown that ammonia can not only pass through the Nafion membrane, but can also be absorbed and interact with the membrane, producing unfavorable ammonia quantification and poor long-term stability operation [9,10,11]. While these effects may be limited by reducing the membrane thickness, possible contaminants already existing in the membrane may still be an issue. Another crucial issue to be taken into consideration is the interaction not only of ammonia with the electrolyte, as already reported in the literature [9], but also of Nafion with the electrolyte, since it may interact with ions present in the electrolyte by cation exchanging. It is therefore important to explore other appropriate membranes to replace Nafion and to obtain accurate NRR tests. As a substitute for Nafion, a salt bridge [12], or a monolayer polypropylene (PP) separator membrane (Celgard) [13], has been proposed. However, the structure and preparation of the salt bridge are complex, and longtime experimental data are not available. Zirfon^®^ is a porous composite diaphragm composed of a polysulfone network and ZrO_2_ as the inorganic filler [14]. Since it offers high chemical stability in concentrate KOH solutions (30%), even at elevated temperatures (together with good structural stability and low resistance), it has been proposed as a separator for alkaline water electrolysis. To our knowledge, there are no literature data concerning the use of Zirfon as a separator for NRR. In this paper, we evaluated the ability of Nafion to absorb and release ammonia and compared it to Zirfon.

Since many NRR experiments are performed in Na_2_SO_4_ solutions, we have investigated this electrolyte as a good candidate for ammonia synthesis and compared it to the case of pure water. The porous Zirfon diaphragm exhibits good hydrophilicity and very small absorption and release of ammonia, therefore representing a promising solution as a separator in the H-type cell for NRR catalyst evaluations.

## 2. Materials and Methods

### 2.1. Materials

Sodium sulfate anhydrous (Na_2_SO_4_, ≥99.0%) was purchased from Carlo Erba and used to prepare electrolytes with a concentration of 0.1 M. Sodium citrate dihydrate (≥99.0%), salicylic acid (99.5%), sodium nitroferricyanide de-hydrate (99.0%), ammonium chloride (NH_4_Cl, 99.99%) and sodium hypochlorite solution (NaClO, 6−14%) were bought from Alfa Aesar and used to measure the ammonia in the electrolyte via the indophenol method. All the solutions were prepared with ultrapure water (MilliQ).

Nafion 117 membranes (N117), purchased from Dupont, were used for the absorption test, with a nominal thickness of 182 µm. The Zirfon Perl (ZP) separator was purchased from Agfa.

### 2.2. N117 Membrane Protonation and ZP Cleaning

N117 membranes were protonated according to the most common procedure described in [15,16]:

1. Soaked in MilliQ for 1 h at 90 °C;
2. Soaked in H_2_O_2_ 3% wt for 1 h at 90 °C and rinsed in MilliQ at R.T;
3. Soaked in H_2_SO_4_ 0.5 M for 3 h at 90 °C and rinsed in MilliQ at R.T;
4. Soaked in MilliQ for 1 h at 90 °C and finally rinsed in MilliQ at R.T;
5. Finally, the membrane was stored in a closed vial for 1 h.

The ZP membrane was cleaned by thoroughly rinsing it in MilliQ water several times before all experiments.

### 2.3. Ammonia Absorption/Releasing Test

The ammonia absorption tests were performed by immersing the N177 and ZP membranes, which were circular with a diameter of 2 cm, in a volume of 15 mL of 0.1 M Na_2_SO_4_ electrolyte (pH 6.5), or in pure water in a glass container, with an ammonia concentration of 0.1 µg mL^−1^ (0.1 ppm). To follow the absorption of ammonia, aliquots of 1 mL were collected from the solution at regular time intervals (0, 10, 20, 40, 60 and 120 min). The collected samples were analyzed to quantify the ammonia concentration via the indophenol blue method, described further in the paper.

For the releasing test, the membranes were taken out from their solution and gently washed with MilliQ and then soaked in a fresh solution of the same Na_2_SO_4_, or in pure water. As described for the absorption test, aliquots of 1 mL of electrolyte were collected at the same time intervals and analyzed to determine the ammonia release from the membrane.

### 2.4. Ammonia Diffusing Process/Migration from Membrane

The migration test was performed using a small one-pot H-Cell with the compartments separated by the ZP. The cathode cell was filled with 20 mL of 0.1 µg mL^−1^ of ammonium chloride solution, and the anode cell with 10 mL of pure water. Similar to the absorption/release test, aliquots of 1 mL were collected from the solution in both chambers at regular time intervals (0, 10, 20, 40 and 60 min) and then tested via the indophenol method to quantify the ammonia amount. The different amounts of electrolyte in the two compartments were ascribed to the design of the electrochemical cell.

### 2.5. Ammonia Quantification

The amount of ammonia in the solutions was measured based on the indophenol blue method reported by Di Zhu et al., with slight modifications [17]: 1 mL of the electrolyte was mixed with 1 mL of a mixture solution consisting of NaOH (1 M), sodium citrate (5 wt%) and salicylic acid (5 wt%), followed by adding 0.5 mL of a NaClO solution (0.05 M) and 0.3 mL of a sodium nitroferricyanide solution (0.1 wt%). The obtained mixture was kept in the dark for 30 min to prevent the Fe-catalyst photodegradation. The absorbance of the solution was detected at λ = 655 nm through an ultraviolet–visible (UV−vis) spectrophotometer (Bentham PV300).

To calculate the amount of NH_3_ produced, the calibration curve was fitted using standard ammonia chloride solutions, prepared in water or in Na_2_SO_4_ electrolyte solution in the range between 0.15 and 0.015 µg mL^−1^. Typical calibration curves are shown in Figures S1 and S2 for water and Na_2_SO_4_, respectively.

### 2.6. NRR Experiment

The ZP and N117 membranes were adopted as separators in a typical NRR experiment performed into a two-compartment cell (H-cell by Redox.me). The electrochemical measurements were carried out in a three-electrode configuration, with a Pt wire as a counter electrode (CE) and a saturated calomel electrode (SCE, saturated KCl) as a reference. The working electrode was Ni foam covered with Au. The anodic and the cathodic chambers were filled with 8 mL and 20 mL of 0.1 M Na_2_SO_4_, respectively, and Ar or N_2_ gas was fluxed in the cathode chamber. The current–voltage measurements and the chronoamperometry were performed by using a Keithley 2600-Source Current Unit.

## 3. Results and Discussion

In order to evaluate the ability of the membranes to absorb and release ammonia, the NH_3_ concentration was measured in the immersion solution. The corresponding variation in ammonia in µg ($\Delta_{\mu g}$) was calculated by the following equations:

(1) $$\Delta_{\mu g}=17000\cdot \left(M_{t}-M_{t+1}\right)\cdot V_{t+1}$$

(2) $$\mu g_{\left(t\right)}=\mu g_{t}-\Delta_{\mu g\;t}$$

where $M_{t}\;$ is the molar concentration of ammonia present at a specific time, $M_{t+1}$ is the ammonia molar concentration after the time interval, and $V_{t}$ is the solution’s volume present in the vial. In Equation (2), $\mu g_{t}$ is the quantity of ammonia present in the solution at a specific time, and $\Delta_{\mu g\;t}$ is the corresponding quantity variation at the same time.

### 3.1. Membranes in Water Ammonium Solution

**Figure 1:**
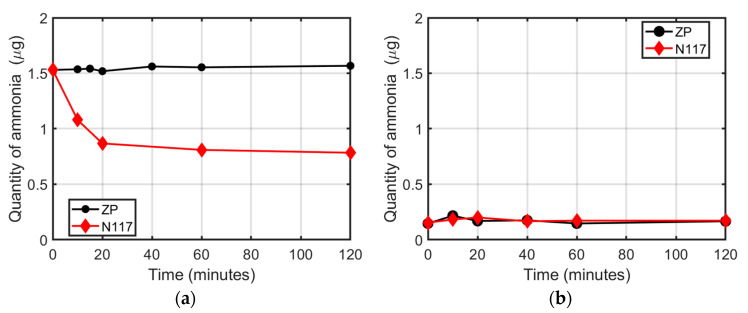
Ammonia quantity as a function of the immersion time: (**a**) in the water NH_4_Cl solution, with N117 or ZP (membrane absorption experiment); (**b**) in pure water after the immersion of N117 or ZP, which had previously absorbed ammonia (release experiment).

Figure 1a shows as red and black symbols the amount of ammonia measured after the immersion of the N117 and ZP membranes, respectively, in 15 mL of the 0.1 ppm NH_4_Cl solution. The starting concentration of 0.1 µg mL^−1^, corresponding to 1.5 µg of ammonia, decreased with time in the case of N117 immersed in the solution. From 0 to 20 min, the N117 membrane rapidly absorbed the ammonium ions present in the solution, which decreased to an amount of 0.86 µg, corresponding to 43% of the starting value. After 120 min, about 49% (0.78 µg) of the ammonia initially present in the electrolyte had been adsorbed by the N117. Conversely, the ZP did not significantly alter the ammonia quantity, which remained near the initial value (±1%), as reported in Figure 1a. The corresponding spectra are shown in Figure S3.

Figure 1b shows the time evolution of the quantity of ammonia in pure water, after the immersion of the same membranes, which had previously absorbed ammonia. The volume of water was the same (15 mL).

Both polymers did not exhibit appreciable ammonia release during the time of the experiment. Moreover, the detected ammonia amount was near the low detection limit of the indophenol blue method technique (0.01 µg mL^−1^) [18]. Under these circumstances, the absorbed ammonium ions in N117 were not released after the immersion in pure water, according to the fact that no ion exchange occurred.

### 3.2. Membranes in Na_2_SO_4_ Ammonium Solution

After performing the first experiment, the same N117 membrane was cleaned by repeating the activation procedure as described in Section 2.2 and rinsed in MilliQ. Just after cleaning, N117 was immersed in a fresh 0.1 M Na_2_SO_4_ electrolyte solution containing 1.5 µg of ammonium ions (0.1 µg mL^−1^ of NH_4_Cl).

**Figure 2:**
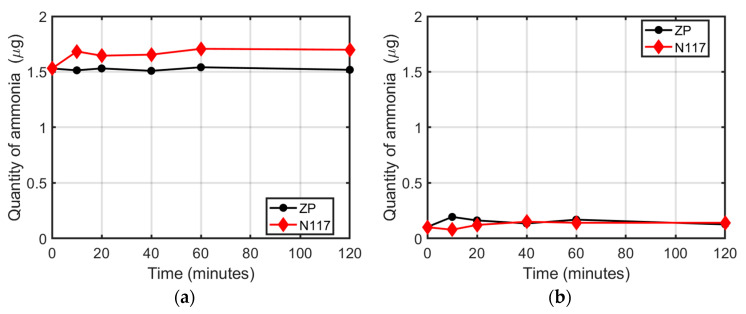
Ammonia quantity as a function of immersion time: (**a**) in the ammoniated electrolyte solution (0.1 Na_2_SO_4_ + 0.1 ppm NH_4_Cl) with N117 or ZP; (**b**) in a 0.1 M Na_2_SO_4_ solution after the immersion of N117 or ZP previously tested.

Figure 2a shows the time evolution of the amount of ammonia in the electrolyte after the immersion of the membranes (N117 or ZP). The ZP did not absorb or release any amount of ammonium in the electrolyte solution, and the quantity remained constant at 1.5 µg, confirming the chemical inertia of ZP against ammonium ions, as already observed in the case of pure water. The N117, instead, showed an initial release of ammonium in the 0.1 M Na_2_SO_4_ ammonium solution during the first 10 min of the absorption experiment, despite the protonation procedure. This confirmed the difficulties of thoroughly removing the NH_4_^+^ cations from the cavities of the N117 with the reported procedure and the capacity of Nafion to absorb ammonia from the environment. After 10 min, the quantity of ammonium ions remained at a constant value of 1.7 µg. The results evidenced the high affinity of N117 with Na^+^ ions. Indeed, sodium cations may exchange with the ammonium ions eventually present inside the membrane and permit its release in the solution, as shown in Figure 2a.

Figure 2b shows the release of ammonium ions as a function of time, produced by the same membranes immersed in a fresh electrolyte solution (ammonia-free 0.1 M Na_2_SO_4_). As already reported in the case of pure water (Figure 1b), both the N117 and ZP did not alter the initial concentration.

### 3.3. Absorption in Water Ammonium Solution and Release in Na_2_SO_4_

**Figure 3:**
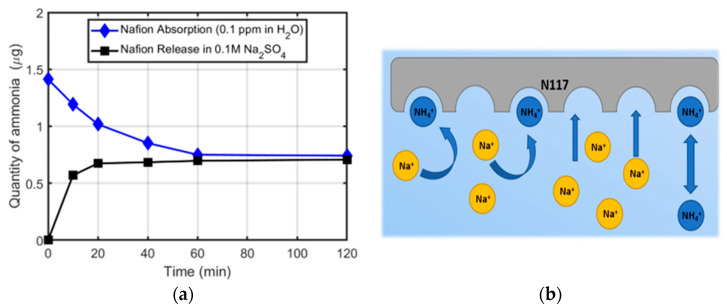
Ammonia quantity as a function of time: (**a**) in NH_4_Cl water solution with N117 (black squares), and in 0.1 M Na_2_SO_4_ solution with N117 loaded with ammonia (blue circles); (**b**) simplified scheme of the interactions between ions in solution and N117 membranes in the case of a 0.1 M Na_2_SO_4_ solution.

To better understand the role of Na^+^ cations in the exchange equilibrium, we repeated the same experiments with the N117 by performing the absorption test in ammonium water solution and the release in 0.1 M Na_2_SO_4_ ammonia-free electrolyte. Indeed, it has been shown that, even when kept in MilliQ water, a significant amount of ammonia can be measured in Nafion after 2 days [19]. Therefore, such an experiment may reproduce a typical situation in which Nafion contaminated by the environment is employed for NRR in a Na_2_SO_4_ ammonia-free solution. Figure 3a shows the time profile of the absorption of ammonium ions by N117 in water and the following release after immersion in the Na_2_SO_4_ solution. The corresponding spectra are reported in Figure S4. A burst release profile was evident, with the ammonium ions released during the first 10 min. This is because monovalent sodium cations are a competitor ligand for the sulfonate sites inside the Nafion membrane, as depicted in Figure 3b. In this circumstance, the ammonium ions absorbed by Nafion during the storage time can be easily released in the Na_2_SO_4_ solution through the ion exchange mechanism. Furthermore, the ion exchange in the N117 affects the conductivity of the membrane. In the works of Thiago Lopes et al. [20] and Tatsuhiro Okada et al. [21], it is effectively explained how the cation interference reduces the conductivity and the water uptake of the Nafion-based membranes. Moreover, once the sodium cations are introduced into the hydrophilic cavity of the Nafion and are strongly bound with the sulphonic pendant, they are difficult to remove [22]. On the contrary, either in pure water or in Na_2_SO_4_, the ammonia absorption by ZP membranes is negligible, as stated before. As an advantage, the channels of ZP do not interact with the ions, and the conductivity is just slightly affected and remains similar to that of the protonated N117 [23].

### 3.4. Ammonium Ion Migration through the ZP Membrane

**Figure 4:**
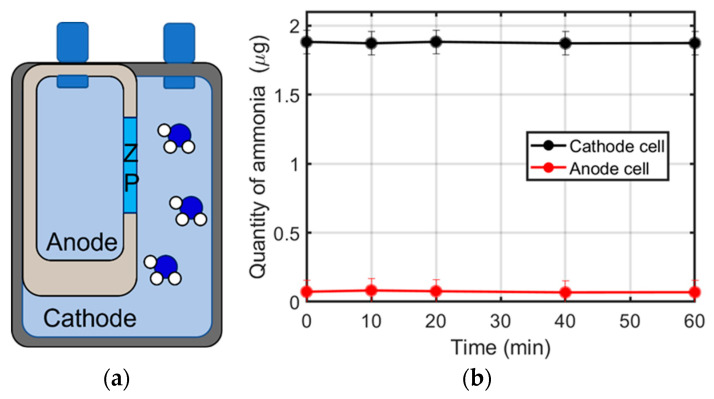
Scheme of the experimental setup: H-cell with Zirfon as separator and ammonia in the cathode compartment (**a**). Ammonia quantity in water solution in the two electrochemical chambers as a function of time (**b**).

In order to evaluate the effectiveness of ZP as an ammonium ion separator, we positioned the ZP as a membrane in an H-cell, as shown in Figure 4a, and filled the cathode side with a water ammonium solution containing 1.88 µg of ammonia. The anode side was filled with pure water. Figure 4b shows the effect of the ZP membrane against the ammonium ion migration between the two chambers. The quantity of NH_4_^+^ ions at the cathode side remained constant for the whole time of the experiment. Results were similar for the anode chamber, where the starting amount was 0.07 µg. Negligible variation in ammonia concentration, near the detection limit of the indophenol method, was observed on both sides from 10 to 60 min. These results confirmed the inertia and the impermeability of the ZP membrane to the ammonium ions, which makes it a suitable alternative to the most commonly used N117 membranes, especially for the NRR experiments.

An important characteristic of membrane separators is their mechanical stability under experimental conditions. Ren Y. et al. reported that the N117 internal structure changes and presents roughness and loosening after use in ammonia-based solutions [12]. Therefore, we analyzed the ZP membrane by optical microscopy and by SEM before and after experiments to verify possible damage or structural modifications, as depicted in Figure S5. There were no scratches or holes, as previously reported for N117, but only a change in the surface roughness was observed [12].

The results can be effectively explained by considering the different transport mechanisms occurring in the two types of membranes. For the Nafion, there are two models proposed in the literature, both based on the assumption that the polymer chains aggregate and create channels whose walls are covered by sulfonic acid functional groups. In particular, according to the first model, the hydrolyzed ionic sites SO_3_^−^ and H_3_O^+^ promote the so-called proton-hopping mechanism [24]. The second model, the vehicular mechanism, relies on the generation of the electro-osmotic flow of the hydrated protons through the free volumes within polymeric chains, and water is the vehicle through which transport takes place [25,26]. Some authors report that both NH_3_ and NH_4_^+^ interact with the sulfonic acid functional groups of Nafion; as a result, the ions absorbed in the membrane are expected to interfere with NRR experiments [19,27,28].

Zirfon is a polysulfone material enriched with ZrO_2_ and developed to avoid the mixing of hydrogen and oxygen gases in alkaline electrolysis [29]. The addition of inorganic fillers to a polymer membrane is a strategy adopted to obtain a composite structure with enhanced filtration properties and a porous structure with high wettability where ions can freely pass through [30]. In these types of membranes, the principal transport mechanism is ruled by an electro-osmotic flow of electrolyte ions from the anode to the cathode. Therefore, the transport takes place without interaction with the membrane polymer chains, due to the chemical inertness of polysulfone [31]. Given this transport mechanism, the ZP membrane is immune to interactions with ammonia, and therefore may allow more reliable results during NRR experiments.

### 3.5. NRR Experiment

**Figure 5:**
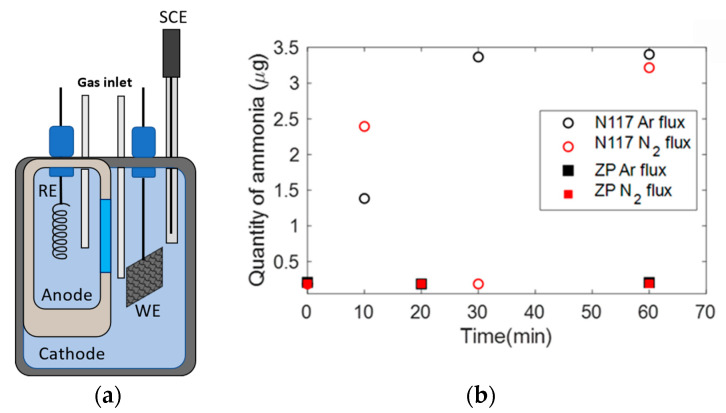
Scheme of the experimental setup for the NRR (**a**). Ammonia quantity in electrolyte solution in the cathodic chambers under Ar or N_2_ flux (**b**).

In order to further verify the feasibility of the ZP for NRR experiments and compare it to Nafion, we adopted an H-cell with anode and cathode compartments separated by either N117 or ZP, with 0.1 M Na_2_SO_4_ electrolyte, as depicted in Figure 5a. Figure 5b shows the ammonia amount in the cathode compartment of the H-cell measurement as a function of time under Ar or N_2_ flux. Data shown in Figure 5b were obtained without applying any voltage. The contrast is evident: when we adopted N117 as a membrane, the ammonia measured in the electrolyte varied as a function of time, increasing or fluctuating from high to low values, due to the release and absorption of ammonia present in the Nafion, kept under environmental conditions. When using ZP, the ammonia in the electrolyte did not change with time and remained stable in the initial low value.

**Figure 6:**
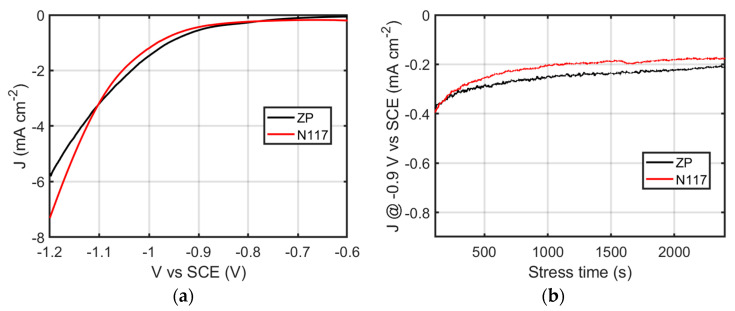
Linear sweep voltammetry performed in an H-cell with anode and cathode compartments separated by Nafion (N117) or Zirfon membranes (**a**). Chronoamperometry of the Au-loaded Ni foam performed at −0.13 V vs. RHE (**b**).

Linear sweep voltammetry measurements were performed by adopting Ni foam covered with gold as a cathode, using N117 or ZP. The comparison, obtained under N_2_ flux, is shown in Figure 6a. The current–voltage characteristic was not affected by the adopted membrane.

The chronoamperometry in N_2_ performed with Nafion or ZP at a constant voltage of −0.9 V vs. SCE is shown in Figure 6b. The electrochemical response of the electrode under test was not strongly affected by the type of membrane. On the contrary, the evaluation of the produced ammonia after 1 h chronoamperometry was strongly affected by the adopted membrane. Indeed, in order to avoid external contamination, the protocol for ammonia measurements requires that the ammonia amount produced during the NRR experiment in N_2_ flux is subtracted by the amount of ammonia initially present in the cell at the same conditions, or by the amount of ammonia produced during chronoamperometry, using Ar flux as the control. When using Nafion, the ammonia release and absorption occurring either under nitrogen or Ar flux and with or without applied potential prevents any reliable evaluation. On the contrary, when using Zirfon, the produced ammonia can be evaluated by adopting the protocol suggested in the literature [5]. The produced amounts under Ar and nitrogen flux are reported in Figure S6.

## 4. Conclusions

We investigated the interaction of a Nafion N117 membrane and a Zirfon membrane with ammonia and with an electrolyte by performing ammonia ion release measurements in pure water and in a Na_2_SO_4_ aqueous solution. In the case of N117, the NH_4_^+^ ions were easily absorbed by the membrane in an aqueous solution free of other cations. The amount of ammonia inside the N117 reached 50% of the total amount in the solution in two hours. The release started when the concentration of other cations, in our case sodium, was present in the aqueous medium in a quantity higher than the ammonium. In this circumstance, the sodium ions replaced the ammonium ions bound with the sulphonate residues inside the membrane, which were then released into the electrolyte solution. Therefore, the use of N117 as a separation membrane in Na_2_SO_4_ can result in a misinterpretation of the initial and final ammonia quantification and a consequent non-reproducibility of the experiments. On the contrary, the ZP membrane did not show significant ammonium ion absorption and consequent release in all the tested conditions. Considering its inertia towards ammonia, ZP seems to be a more suitable membrane for NRR electrochemical experiments.

## Data Availability

The data of this study are available from the corresponding author upon reasonable request.

## Supplementary Materials

The following supporting information can be downloaded at: https://www.mdpi.com/article/10.3390/membranes12100969/s1, Figure S1: (a) UV-Vis absorbance spectra for the indophenol procedure with various NH_4_^+^ concentration ranging from 0.015 µg mL^−1^ to 0.015 µg mL^−1^ in 0.1 M Na_2_SO_4_ solution. (b) The corresponding calibration curve; Figure S2: (a) UV-Vis absorbance spectra for the indophenol procedure with various NH_4_^+^ concentration ranging from 0.015 µg mL^−1^ to 0.015 µg mL^−1^ in MilliQ solution. (b) The corresponding calibration curve; Figure S3: UV-Vis absorbance spectra time evolution for the ammonium ions absorption test in ammoniated water solution; (a) N117 membrane (b) ZP; Figure S4: UV-Vis absorbance spectra time evolution for the ammonium ions absorption test for N117 in (a) ammoniated water solution and the release experiment in (b) 0.1 M Na_2_SO_4_ solution; Figure S5: Optical micrographs at low magnitude of the same samples (a) before and (b) after dipping in 1 ppm ammonia solution for 24 h. SEM micrographs of Zirfon (c) before and (d) after the same dipping test. Although the roughness is slightly changed, there are no holes, scratches or any type of visible damage which would compromise the separation of the electrolytic chambers; Figure S6: Quantity of ammonia measured in the cathode compartment of the H-cell using ZP as separator, at the starting of the NRR experiment and after 1 h chronoamperometry under Ar or N_2_ flux.
