# Supplementary material for: Role of the Membrane Transport Mechanism in Electrochemical Nitrogen Reduction Experiments
Source: Membranes (Basel). 2022 Oct 2;12(10):969. doi: 10.3390/membranes12100969 (PMC9607467; doi:10.3390/membranes12100969)
Supplement: Supplementary file 1 [file membranes-12-00969-s001.zip › membranes-1905186-supplementary.pdf]

membranes

MDPI

Supplementary Material for:

Role of the Membrane Transport Mechanism in Electrochemical Nitrogen Reduction Experiments

Marco Leonardi [1,2,*], Giuseppe Tranchida [1,3] , Roberto Corso[1,2], Rachela G. Milazzo [1], Salvatore A. Lombardo [1] and Stefania M. S. Privitera [1]

[1] National Research Council Institute for Microelectronics and Microsystems (CNR-IMM), Strada VIII, 5, 95121 Catania, Italy
[2] Department of Physics and Astronomy, University of Catania, Via Santa Sofia, 64, 95123 Catania, Italy
[3] Department of Chemical Sciences, University of Catania, Viale Andrea Doria, 6, 95125 Catania, Italy
* Correspondence: marco.leonardi@imm.cnr.it

1. Supplementary Information

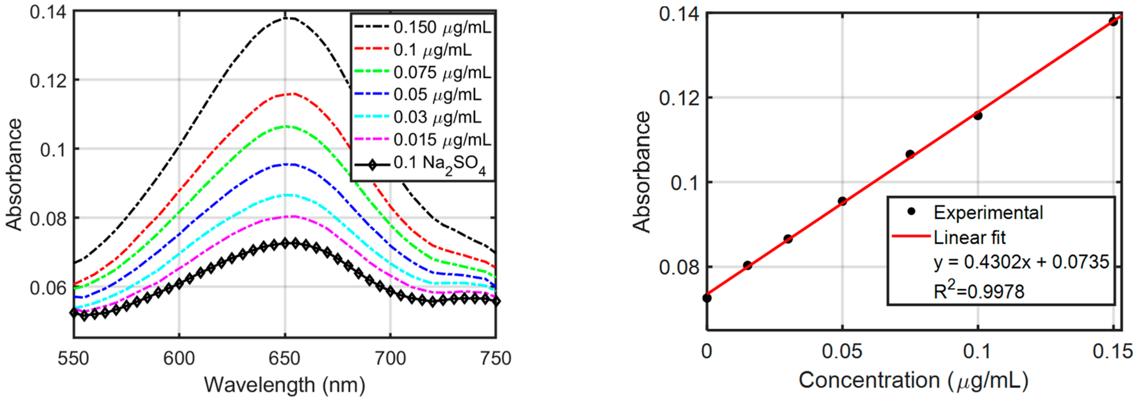

(**a**) (**b**)

Figure S1. (**a**) UV-Vis absorbance spectra for the indophenol procedure with various NH_4^+ concentration ranging from 0.015 μg mL^{-1} to 0.015 μg mL^{-1} in 0.1 M Na_2SO_4 solution. (**b**) The corresponding calibration curve.

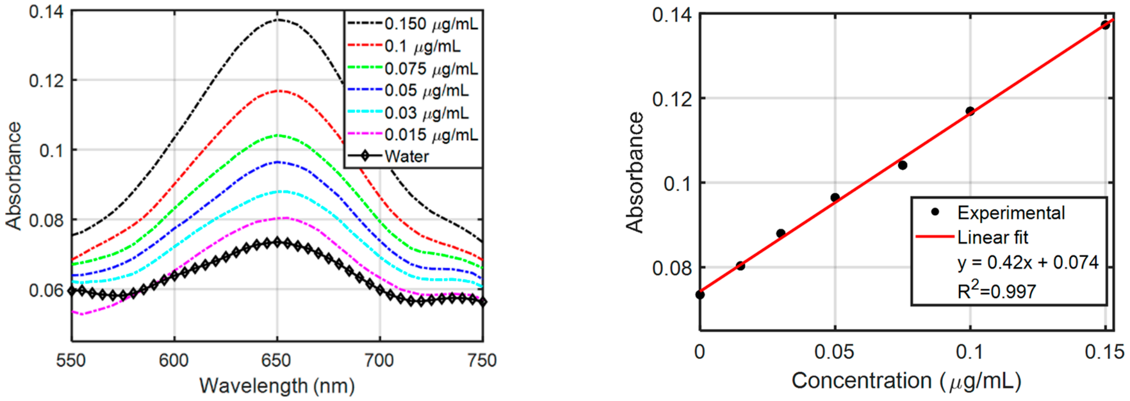

(**a**) (**b**)

Figure S2. (**a**) UV-Vis absorbance spectra for the indophenol procedure with various NH_4^+ concentration ranging from 0.015 µg mL^{-1} to 0.015 µg mL^{-1} in MilliQ solution. (**b**) The corresponding calibration curve.

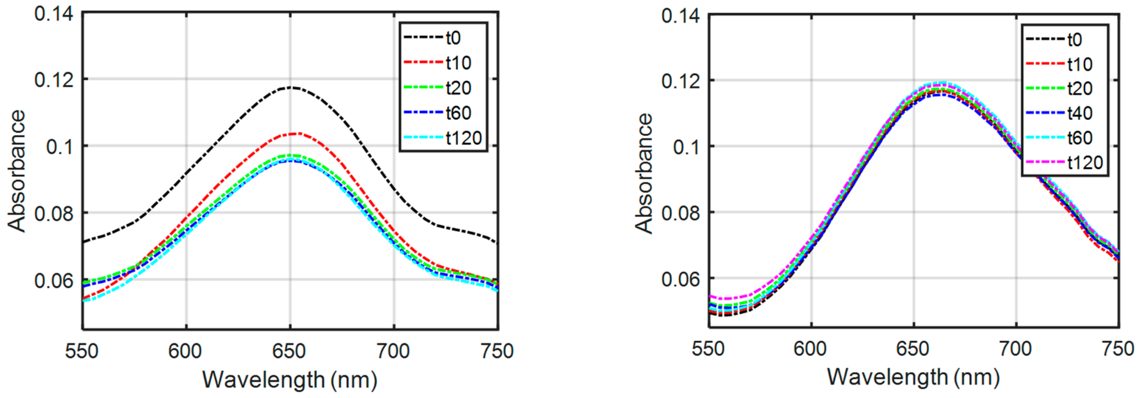

(**a**) (**b**)

Figure S3. UV-Vis absorbance spectra time evolution for the ammonium ions absorption test in ammoniated water solution; (**a**) N117 membrane (**b**) ZP.

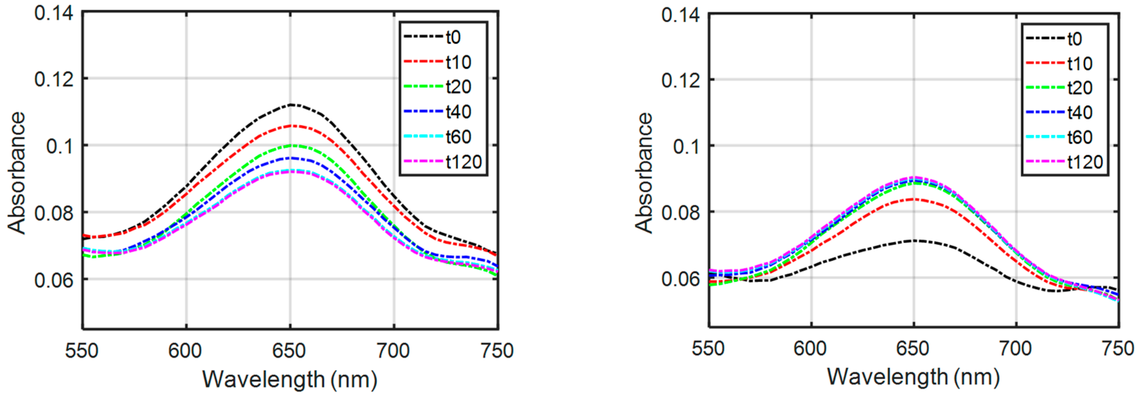

(**a**) (**b**)

Figure S4. UV-Vis absorbance spectra time evolution for the ammonium ions absorption test for N117 in (**a**) ammoniated water solution and the release experiment in (**b**) 0.1 M Na_2SO_4 solution.

The morphology has been investigated Optical microscopy, to observe a wide area, and by Scanning Electron Microscopy (SEM) using a ZEISS SUPRATM 35.

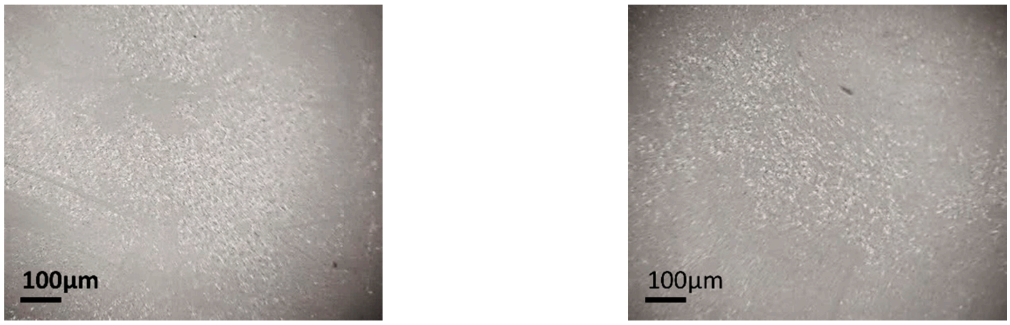

(**a**) (**b**)

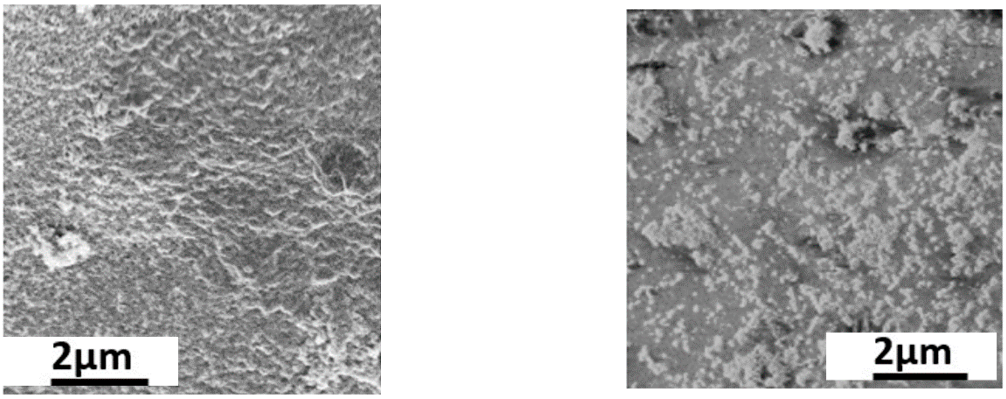

(**c**) (**d**)

Figure S5. Optical micrographs at low magnitude of the same samples (**a**) before and (**b**) after dipping in 1 ppm ammonia solution for 24 hours. SEM micrographs of Zirfon (**c**) before and (**d**) after the same dipping test. Although the roughness is slightly changed, there are no holes, scratches or any type of visible damage which would compromise the separation of the electrolytic chambers.

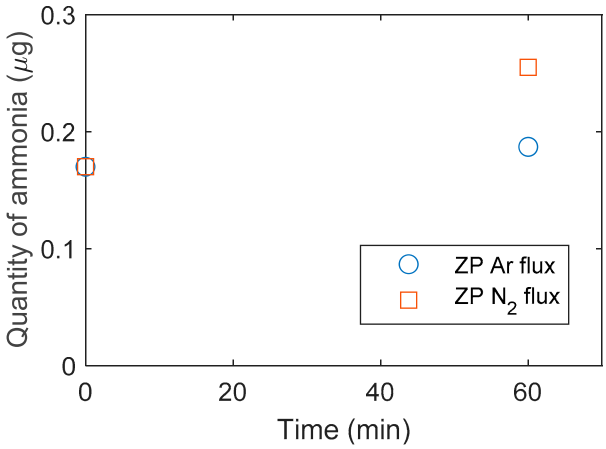

Figure S6. Quantity of ammonia measured in the cathode compartment of the H-cell using ZP as separator, at the starting of the NRR experiment and after 1 h chronoamperometry under Ar or N_2 flux.
